# Exploring the construct validity of the Patient Perception Measure – Osteopathy (PPM-O) using classical test theory and Rasch analysis

**DOI:** 10.1186/s12998-015-0055-x

**Published:** 2015-03-02

**Authors:** Jane Mulcahy, Brett Vaughan

**Affiliations:** Centre for Chronic Disease Prevention & Management, College of Health & Biomedicine, Victoria University, Melbourne, Australia; Institute of Sport, Exercise & Active Living, Victoria University, Melbourne, Australia; School of Health & Human Sciences, Southern Cross University, Lismore, Australia

## Abstract

**Background:**

Evaluation of patients’ experience of their osteopathic treatment has recently been investigated leading to the development of the Patient Perception Measure – Osteopathy (PPM-O). The aim of the study was to investigate the construct validity of the PPM-O.

**Methods:**

Patients presenting to osteopathy student-led teaching clinics at two Australian universities were asked to complete two questionnaires after their treatment: a demographic questionnaire and the PPM-O. Confirmatory factor analysis (CFA) and Rasch analysis were used to investigate the construct validity of the PPM-O.

**Results:**

Data from the present study did not fit the *a-priori* 6-domain structure in the CFA. Modifications to the 6-domain model were then made based on the CFA results, and this analysis identified two factors: 1) Education & Information (9 items); and 2) Cognition & Fatigue (6 items). These two factors were Rasch analysed individually. Two items were removed from the Cognition & Fatigue factor during the analysis. The two factors independently were unidimensional.

**Conclusions:**

The study produced a 2-factor, 13-item questionnaire that assesses the patients’ perception of their osteopathic treatment using the items from a previous questionnaire. The results of the current study provide evidence for the construct validity of the PPM-O and the small number of items makes it feasible to implement into both clinical and research settings. Further research is now required to establish the measures’ validity in a variety of patient populations.

**Electronic supplementary material:**

The online version of this article (doi:10.1186/s12998-015-0055-x) contains supplementary material, which is available to authorized users.

## Background

A common concern of clinicians and clinical educators working directly with patients is the significant variance in individual treatment efficacy of patients. The proposed causes for this variability in patients’ experiences of their health encounters are complex and multidimensional. Demographic factors such as: gender, age, social gradient [1], education, ethnicity and geographic location have all been identified as factors that affect general health and disease status [2], as well as access to, and utilisation of, treatments and health services [2]. While nuances of the clinician and the clinical environment contribute to aspects of treatment outcome such as patient satisfaction [3], the patients’ beliefs about their health and wellbeing, their illness or disease and expectations of treatment would also appear to have a significant effect [4,5].

### Patient experience and expectations

Research investigating patients’ experiences during, and as a result of, their treatment has tended to focus on the: patient-therapist interaction [6,7], clinical environment [8], satisfaction with treatment [9], and, efficacy of treatment outcomes [7,10]. The patients’ physical experience of their treatment (i.e. sensations that the patient experiences during or after their treatment) are seldom described in manual therapy research. This aspect of the patients’ experiences of a treatment requires further exploration to develop a more global picture of the patient experience during and after their consultation.

Recently Cross et al. [4] used a qualitative approach to investigate patients’ expectations of osteopathic treatment in private United Kingdom practices and concluded these expectations are primarily related to the patient-therapist interaction. Further, patients identified professional expertise and customer service as expectations of osteopathic treatment. Drawing on this work, Leach et al. [11] used a quantitative approach to identify patient expectations of their osteopathic care. The top three aspects of care highlighted by patients in this study were the ability to ask questions of the practitioner, active listening and respect. Again, the focus was very much on the patient-therapist interaction. Although, these two studies provide valuable insights into what patients expect from an osteopathic treatment, patients’ cognitive, emotional and sensory responses to osteopathic treatment have not previously been established or included in commonly utilised patient reported outcomes measures (PROMs). Previous work by Mulcahy & Vaughan [12] investigated the patient-reported sensory experiences of Osteopathy in the Cranial Field (OCF) treatment. However these sensory experiences have not been validated in patients receiving general osteopathic treatment.

The Patient Perception Measure – Osteopathy (PPM-O) was developed to enhance clinicians and clinical educators understanding of what patients perceive during osteopathic treatment. Items for inclusion in the PPM-O were based on those used in a previous study to explore patient perception of OCF [12,13].

### Confirmatory factor analysis

The CFA was used to determine if the data fitted the 6 domains identified by Mulcahy et al. [13]. CFA produces a variety of fit statistics indicating how well the data collected fits the proposed *a-priori* factor structure [14]. A range of fit statistics should be generated because each statistic has different measurement properties [15,16]. The chi-square statistic is used to report the fit of the data to the model and *p*-values less then 0.05 indicate a fit [17]. Whilst there is no agreement as to which type of fit statistics should be presented, in the current study the authors present a range of statistics to provide the reader with a more comprehensive representation of the data fit. Fit statistics in the present study were in line with those suggested by DiStefano & Hess [15] and included: the goodness of fit index (GFI), comparative fit index (CFI), normed fit index (NFI), Tucker-Lewis index (TLI), root mean square residual (RMR) and the root mean square error of approximation (RMSEA). The use of these fit statistics is also supported by other authors [17,18] and ensures that a range of global fit and relative fit indices are presented [15].

### Rasch analysis

Rasch analysis is part of the modern test theory (MTT) statistical technique group and is widely used in the development and analysis of questionnaires and measures. The approach was developed by Dutch mathematician George Rasch [19] and fit of the data to the Rasch model is the desired outcome of the analysis [20]. Rasch analysis is sample-independent compared to the sample-dependent analyses in classical test theory. In Rasch analysis the data is fitted to a mathematical model to determine if all respondents are responding to each item in a manner dictated by the Rasch model. A range of statistics related to the interaction between the questionnaire items and the person responses (item-trait interaction) is generated. The item-trait statistics demonstrate the overall fit of the items and persons to the Rasch model [21]. This statistic analyses how each item on the PPM-O relates to all other items, and how each person is responding to each item on the PPM-O. These statistics indicate how the responses fit those expected by the Rasch model. A Bonferonni-adjusted non-statistically significant chi-square indicates an overall fit of all persons and items to the Rasch model [21]. Rasch model item and person fit is indicated by a fit residual standard deviation (SD) of ± 1.5. Fit residual SDs outside of this range suggests that issues exist with the model fit of the items and/or persons. A Person Separation Index (PSI) is also generated to indicate the internal consistency of the questionnaire being analysed and is interpreted in the same way as Cronbach’s alpha [22].

Fit of the individual items to the Rasch model is analysed to ascertain whether misfitting items are impacting upon the overall model fit. Poor individual item fit is indicated by a fit residual of ± 2.5 and/or a statistically significant chi-square probability [23]. In the case of the PPM-O it may be that the probability of a patient selecting a particular response on the Likert-type scale is not equal for all possible responses on the scale – this is referred to as a disordered threshold. The threshold is the point at which there is a 50% chance of a person selecting response 1 or response 2 on a scale [21,23]. Where the threshold is disordered, respondents are selecting scale responses in a manner that is not consistent with the trait under investigation. A disordered threshold may also result from persons answering the item having trouble differentiating between the scale responses (i.e. likely, very likely, highly likely). It is possible to rescore the item to resolve the threshold disorder [21,24]. The category probability curves are used to ensure that each response on the scale is being used in an ordered manner. Each response option for the item should have the highest probability of being selected at some point along the person location.

Differential item functioning (DIF) is the investigation of how an item functions with respect to a person factor such as age or gender. In the present study, age, gender, satisfaction with life [12] and meaningful daily activity [12,25] were investigated to see if they had an impact on the way a person answers an item or items on the PPM-O. Each person factor is investigated separately to ascertain the impact of it on the fit of the data to the Rasch model. Where an item demonstrates DIF (through a statistically significant Bonferroni adjusted chi-square probability), it can be removed or recalibrated (e.g. those under 20 years of age can be split from those above 20 years of age).

Misfit of individual persons to the Rasch model is indicated by a fit residual of ± 2.5 [23]. A person is said to misfit when their response to each of the item on a questionnaire, in this case on items on the PPM-O, does not follow the prediction of the Rasch model for how that person should have responded to the item. Misfitting persons can impact on the Rasch model [21] and they will often be removed from further analysis.

The dimensionality of the measure is important because this demonstrates whether it is measuring a single underlying construct [26]. Local dependency is where the response to one item dictates the response to another item [23,26] and this can inflate the PSI [27]. Where local dependency is identified (the PSI decreases) one of the correlating items will need to be deleted. Next, the dimensionality is assessed using a Principal Components Analysis (PCA). The PCA is used to generate the ‘Rasch factor’ (factor 1) and display the positively and negatively loaded items. These items are then analysed using a paired t-test to examine whether the positive and negative loaded items are statistically significantly different [21]. Where no statistically significant difference exists, the questionnaire is thought to be unidimensional [21].

### Study aim

The aim of the present study is to explore the construct validity of the Patient Perception Measure - Osteopathy (PPM-O) using both confirmatory factor analysis and Rasch analysis.

## Methods

This study was approved by the Victoria University (VU, Melbourne, Australia) and Southern Cross University (SCU, Lismore, Australia) Human Research Ethics Committees.

### Participants

Patients attending the student-led osteopathy teaching clinics at VU and SCU were invited to participate in the study. At the conclusion of their treatment, patients were invited to complete the PPM-O questionnaire by the reception staff. An Information to Participants sheet was provided to each potential participant and consent to participate was implied by completing the questionnaire. Completed PPM-O and demographic questionnaires were placed in a secure box in the reception area and collected by one of the authors weekly. Only the authors had access to the collected data.

### Measure

The Patient Perception Measure – Osteopathy (PPM-O) is based on the items from a previously developed 22-item questionnaire divided into 6 domains based on an *a-priori* theoretical structure [13]. The domains identified were Education & Information, Cognition & Fatigue, Effectiveness of Osteopathic Treatment, Perceived Emotional Responses to Osteopathic Treatment, Perceived Physical Responses to Osteopathic Treatment, and Application of Osteopathic Principles.

Participants were also asked to complete a single-page demographic questionnaire. Items on the demographic questionnaire included age, gender, employment status, current medication usage and whether the participant suffers, or suffered from, one of the seven major illnesses identified by the Australian Institute of Health and Welfare [2]. Participants were also asked about 2 global items; satisfaction with life (SWL) and meaningfulness of daily activity (MDA) [25]. These global items were rated on a Likert-type scale from 0 to 6, anchored at each end. The anchors for SWL were ‘not at all satisfied’ (0) and ‘extremely satisfied’ (6), and the MDA anchors were ‘not at all meaningful’ (0) and ‘extremely meaningful’ (6). Higher scores on these global items indicated greater satisfaction with life and meaningfulness of daily activity respectively. Elements of the demographic data were used to examine the differential item function in the Rasch analysis.

### Data analysis

Data were entered into SPSS Version 21 (IBM Corp, USA) for analysis. The analysis of the PPM-O took place in two stages: 1) confirmatory factor analysis (CFA); and 2) Rasch analysis. The CFA was conducted with AMOS Version 21 (IBM Corp, USA) using the Maximum Likelihood Method approach. The recommended fit statistic cut-off values for each analysis used in the present study are presented in Table 1. As the data were not normally distributed, a bootstrapping procedure was applied for each of the two models (22 item PPM-O & 13-item PPM-O), and 1000 iterations of the data were generated. Data were exported from SPSS to RUMM2030 [28] to perform the Rasch analysis using the Partial Credit Model [21] as the ‘distance’ between the response categories for each item were thought not to be equal. This was confirmed with a statistically significant Likelihood Ratio Test (p < 0.05). Graphical and numerical threshold maps, and graphical category probability curves were produced in addition to the statistical analysis. In the present study, the decision to remove items demonstrating DIF was made *a priori* to make the PPM-O easy to administer and interpret.

**Table 1:** **CFA fit statistics for the two versions of the PPM-O**

**Statistic**	**Recommended value**	**22-item PPM-O**	**13-item PPM-O**
χ2	NA	357.23	130.46
χ2 p-value	<0.05	>0.0001	>0.0001
df	NA	194	64
χ2/df	< or = 2	1.84	2.04
Goodness of fit index (GFI)	> or = 0.9	0.828	0.879
Comparative fit index (CFI)	> or = 0.9	0.841	0.855
Normed fit index (NFI)	> or = 0.9	0.717	0.757
Tucker-Lewis index (TLI)	> or = 0.9	0.811	0.824
Root mean square residual (RMR)	As close to 0 as possible	0.048	0.054
Root mean square error of approximation (RMSEA)	< or = 0.08	0.075 (CI 0.062-0.087)	0.083 (CI 0.062-0.103)

## Results

One hundred and eighty four questionnaires were received however 32 (18%) contained incomplete data and were subsequently removed from the CFA - 152 questionnaires were analysed in the CFA. Data from all 184 questionnaires were entered into RUMM for the Rasch analysis however one questionnaire did not contain enough data to be able to analysed and was removed. One hundred and eighty three responses (n = 183) were analysed in the Rasch analysis.

The mean age of the respondents was 35.8 years (+/− 15.1 years) and 60.5% (n = 92) were female. Employment status was shared between employed (n = 63, 41.4%) and students who were employed (n = 55, 36.2%). Participants were generally satisfied with their life (4.03 +/− 0.73) and found their daily activity moderately meaningful (3.96 +/− 0.78). No participant indicated they were not satisfied with their life or that their daily activity was not meaningful (corresponding to a score of 0). Data were collected related to the seven major Australian illnesses [2], and prevalence of these disorders were: cardiovascular disease (n = 9, 5.9%); cancer (n = 3, 2.0%); mental health disorder (n = 19, 12.5%); diabetes (n = 4, 2.6%); chronic respiratory complaint (n = 13, 8.6%); and the combined arthritis and musculoskeletal complaints (n = 65, 42.8%).

Descriptive statistics for the participant responses to the PPM-O are presented in Table 2.

**Table 2:** **Descriptive statistics for the 22-item Patient Perception Measure – Osteopathy (PPM-O)**

**Item**	**Response options**	**Min**	**Max**	**Mean**	**Std. Dev.**
1. The way that my osteopath explains my osteopathic treatment is	Poor, fair, good, very good, excellent	3	5	4.52	0.57
2. The way my osteopath answers all of my questions is	Poor, fair, good, very good, excellent	3	5	4.59	0.54
3. My osteopath treats me with respect	Never, rarely, sometimes, mostly, always	3	5	4.97	0.21
4. The instructions my osteopath gives me regarding my home exercise program are	Poor, fair, good, very good, excellent	1	5	4.30	0.71
5. Osteopathic treatment has helped my condition	Never, rarely, sometimes, mostly, always	3	5	4.40	0.60
6. The way my management plan was explained to me was	Poor, fair, good, very good, excellent	2	5	4.26	0.71
7. The osteopathic treatment I have received has improved my quality of life	Never, rarely, sometimes, mostly, always	2	5	4.34	0.65
8. As a result of osteopathic treatment, my general health is	Poor, fair, good, very good, excellent	2	5	3.89	0.72
9. During my treatment, the questions my osteopath asked were	Poor, fair, good, very good, excellent	3	5	4.34	0.63
10. After my osteopathic treatment I felt like my whole body was treated rather than just one area	Never, rarely, sometimes, mostly, always	2	5	4.25	0.79
11. Osteopaths at this clinic talk about the body’s ability to heal itself	Never, rarely, sometimes, mostly, always	1	5	3.80	0.94
*12. Osteopathic treatment makes me feel vague*	Never, rarely, sometimes, mostly, always	1	12	2.15	1.24
*13. I cannot focus on tasks after my osteopathic treatment*	Never, rarely, sometimes, mostly, always	1	5	1.86	0.92
14. I feel calmer after my osteopathic treatment	Never, rarely, sometimes, mostly, always	1	5	4.28	0.72
15. Osteopathic treatment makes no difference to my frame of mind	Never, rarely, sometimes, mostly, always	1	5	2.14	1.11
16. How helpful is osteopathic treatment in managing your condition	Poor, fair, good, very good, excellent	2	5	4.22	0.69
*17. I feel sad after osteopathic treatment*	Never, rarely, sometimes, mostly, always	1	5	1.22	0.58
*18. I feel tired after osteopathic treatment*	Never, rarely, sometimes, mostly, always	1	5	2.48	1.04
*19. I am anxious after osteopathic treatment*	Never, rarely, sometimes, mostly, always	1	3	1.16	0.38
*20. I feel alone after osteopathic treatment*	Never, rarely, sometimes, mostly, always	1	4	1.11	0.37
21. I feel less pain after osteopathic treatment	Never, rarely, sometimes, mostly, always	1	5	4.05	0.81
*22. I find it hard to concentrate after my osteopathic treatment*	Never, rarely, sometimes, mostly, always	1	4	1.82	0.88

Note: negatively phrased items are in italics and require rescoring prior to analysis.

Legend – response option scoring.

Poor (1), fair (2), good (3), very good (4), excellent (5).

Never (1), rarely (2), sometimes (3), mostly (4), always (5).

NB these scores are reversed for negatively phrased items.

### Confirmatory factor analysis 1

**Figure 1:**
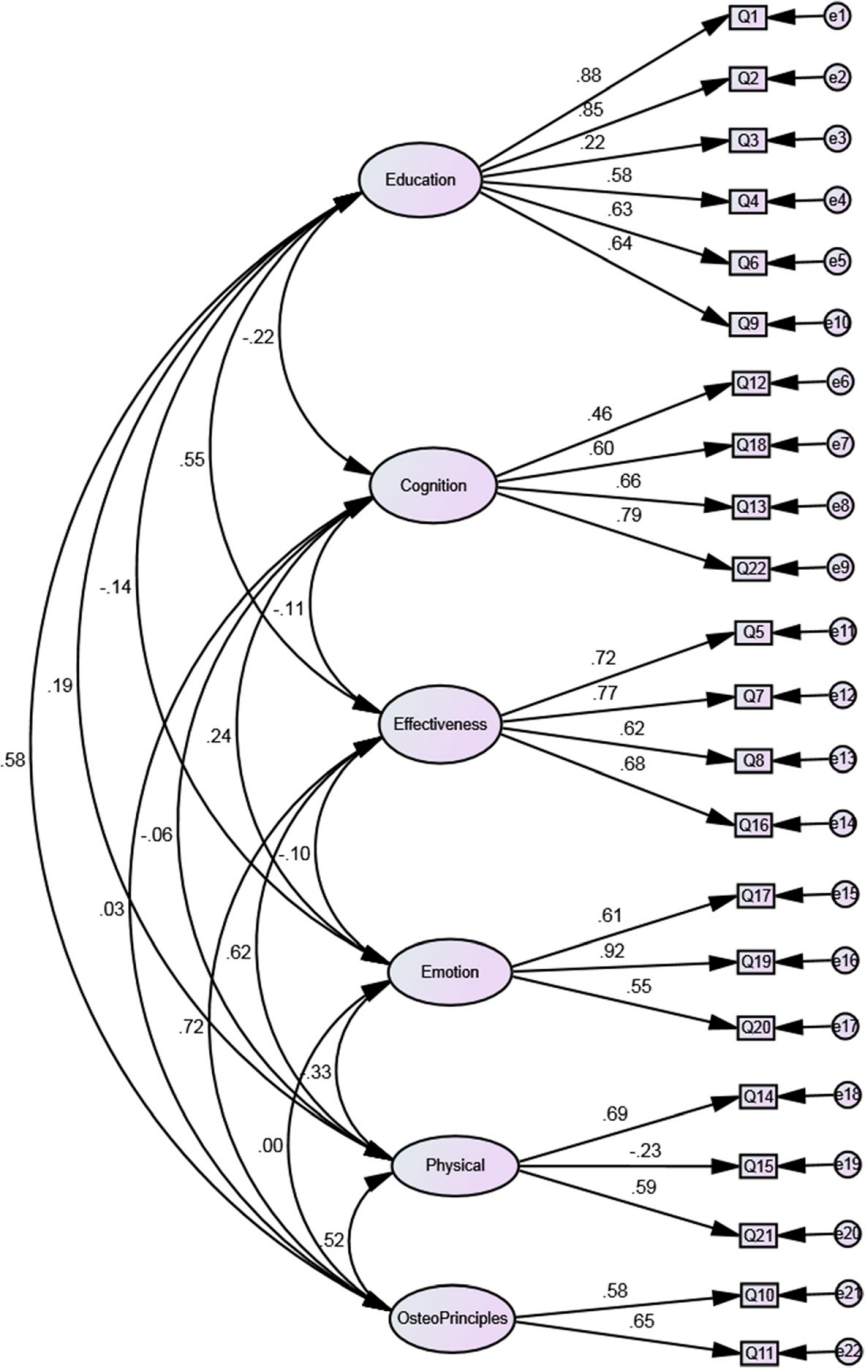
**Path diagram for the 22-item Patient Perception Measure - Osteopathy.**

Data were initially fitted to the *a-priori* 6 domain structure proposed by Mulcahy et al. [13]. The path diagram for this model is presented in Figure 1 and the fit statistics are presented in Table 1. The data did not fit the model as indicated by the statistically significant chi-square probability (p < 0.001) however the GFI was approaching the recommended value.

### Rasch analysis 1

The data for the 22 item PPM-O did not fit the Rasch model (χ2 = 171.95, df = 44, p < 0.0001). The PSI was 0.783 indicating borderline internal consistency. The standard deviation fit residuals for both items (1.22) and persons (0.82) were not greater than 1.5. A poor fit residual (>2.5) was identified for item 18 and statistically significant χ2 values for items 2 and 15, indicating a poor fit of these items to the Rasch model. Disordered thresholds were demonstrated for all items except 5–7, 11 and 15. The completed questionnaire from one person did not contain enough data and was removed, therefore 183 responses were analysed. DIF was identified for SWL and MDA at item 20 (I feel alone after osteopathic treatment). Those participants with low SWL and MDA scores were more likely to endorse this item highly (agree or strongly agree). Assessment of dimensionality indicated that the 22-item questionnaire was not unidimensional.

### PPM-O Modification

#### Confirmatory factor analysis

**Figure 2:**
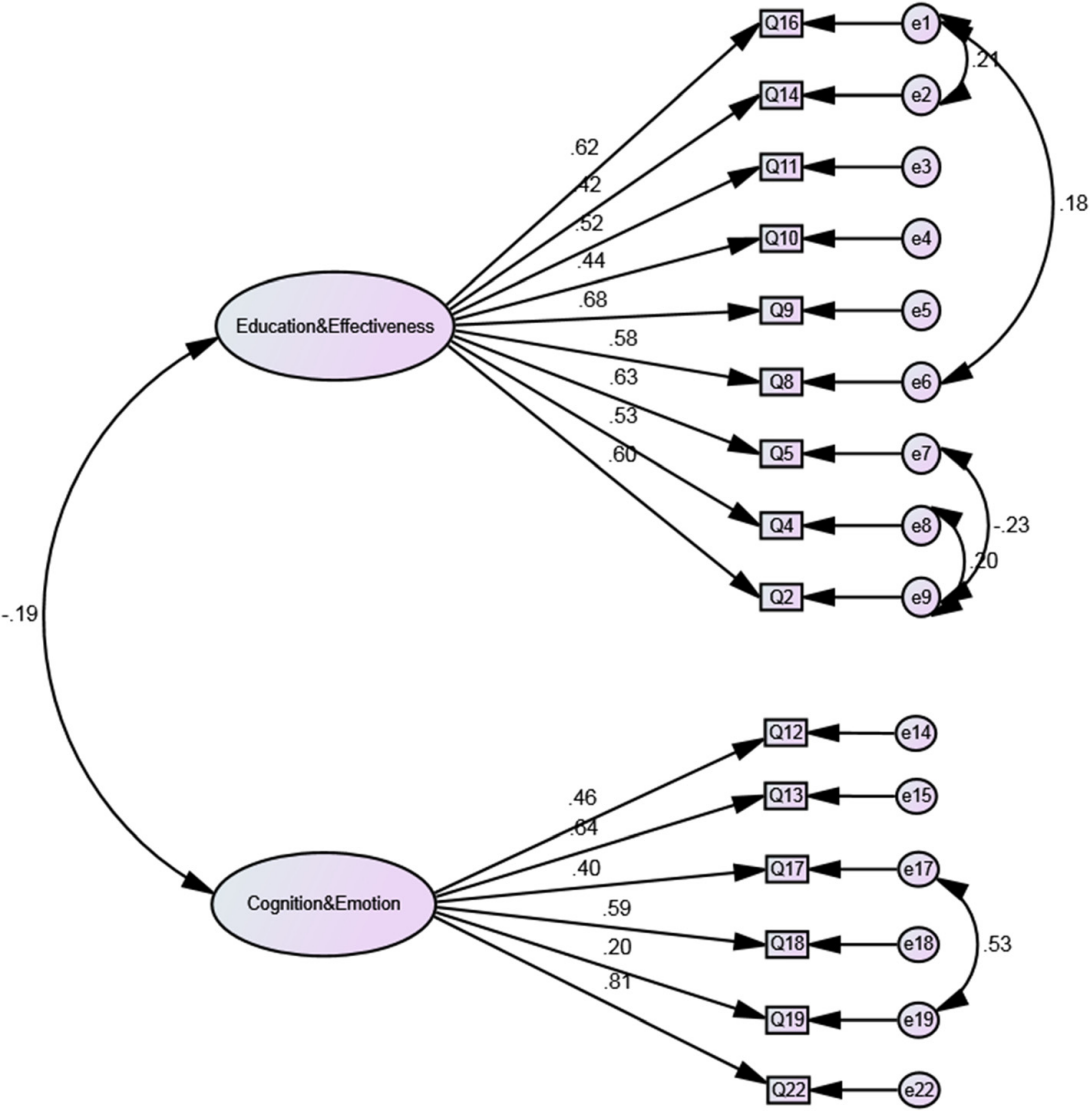
**Path diagram for the 2-factor, 15-item Patient Perception Measure -Osteopathy.**

Given the lack of model fit in the first CFA and the multidimensional nature of the 22-item PPM-O, confirmed through the initial Rasch analysis, the CFA model was modified to establish a multifactorial structure. Item covariances were analysed in order to modify the 22-item PPM-O. An item was removed if the covariance with another item was greater than 10 or did not fit onto a factor. Figure 2 demonstrates the correlation between each of the 6 domains. Strong relationships were identified between the Education, Effectiveness, Physical and Osteopathic Principles factors. The items in these domains were combined into a single factor called Education & Effectiveness. The items remaining in the Cognition and Emotion factors were combined to form the Cognition & Fatigue factor. This process produced a 2-factor, 15 item version of the PPM-O (Figure 2).

### Rasch analysis

The revised 2-factor, 15-item PPM-O was Rasch analysed. As two factors had been identified, they were independently analysed in order for each factor to fit the Rasch model.

#### Rasch analysis of the education & effectiveness factor

**Figure 3:**
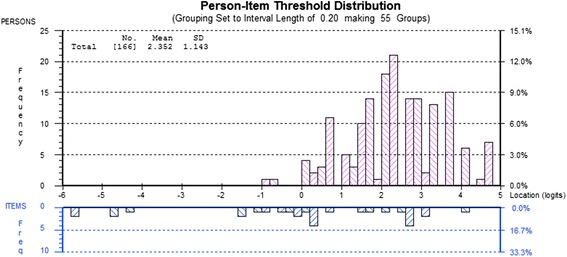
**Person-item map for the Education & Effectiveness factor.**

The Education & Effectiveness factor demonstrated fit to the Rasch model (χ2 = 35.47, df = 18, p = 0.008). The PSI was 0.763. The fit residual SD for items was 0.77 and 0.95 for persons. None of the items demonstrated statistically significant chi-square probabilities or fit residual SDs. Disordered thresholds were observed for all items except 8, 9 and 16 (Additional file 1). There were 15 misfitting persons (out of 183 responses) and none of the items demonstrated DIF for any of the person factors. In order to achieve model fit, a number of modifications were made. Items 2, 4, 5, 9, 11, and 14 were rescored (Additional file 1) and this resolved the disordering for all items. Eighteen misfitting persons were removed from the analysis - these persons were not significantly different from the analysed persons with regard to demographics. All items demonstrated ordered thresholds and there was no DIF for any item. There were no residual correlations. The PCA and subsequent paired t-test of the positively and negatively loading items on the Rasch factor were statistically significant indicating the factor was unidimensional. With the item rescoring, the possible total score for this factor is 39. The mean person-item distribution for this factor is 2.35 (Figure 3). The item fit statistics are presented in Table 3.

**Table 3:** **Item fit statistics for the 2-factor, 13-item Patient Perception Measure – Osteopathy (PPM-O13)**

	**Response options**	**Location**	**Fit residual**	**Chi-square**	**Probability**
**Education & effectiveness**					
1. The way my osteopath answers all of my questions is	Poor, fair, good, very good, excellent	−1.307	−1.157	9.672	0.007
2. The instructions my osteopath gives me regarding my home exercise program are	Poor, fair, good, very good, excellent	0.581	0.776	3.696	0.157
3. Osteopathic treatment has helped my condition	Never, rarely, sometimes, mostly, always	−1.050	−0.071	3.230	0.198
4. As a result of osteopathic treatment, my general health is	Poor, fair, good, very good, excellent	−0.136	−0.091	0.300	0.860
5. During my treatment, the questions my osteopath asked were	Poor, fair, good, very good, excellent	−1.024	−0.608	7.443	0.024
6. After my osteopathic treatment I felt like my whole body was treated rather than just one area	Never, rarely, sometimes, mostly, always	0.495	1.029	4.701	0.095
7. Osteopaths at this clinic talk about the body’s ability to heal itself	Never, rarely, sometimes, mostly, always	1.828	0.863	0.485	0.784
8. I feel calmer after my osteopathic treatment	Never, rarely, sometimes, mostly, always	0.917	0.853	4.371	0.112
9. How helpful is osteopathic treatment in managing your condition	Poor, fair, good, very good, excellent	−0.303	−0.269	2.031	0.362
**Cognition & Fatigue**					
*10. Osteopathic treatment makes me feel vague*	Never, rarely, sometimes, mostly, always	−0.146	0.913	3.192	0.202
*11. I cannot focus on tasks after my osteopathic treatment*	Never, rarely, sometimes, mostly, always	−0.355	−1.429	2.966	0.226
*12. I feel tired after osteopathic treatment*	Never, rarely, sometimes, mostly, always	−0.872	1.225	3.126	0.209
*13. I find it hard to concentrate after my osteopathic treatment*	Never, rarely, sometimes, mostly, always	1.373	−0.745	2.536	0.281

Note: negatively phrased items are in italics and require rescoring prior to analysis.

Legend – response option scoring.

Poor (1), fair (2), good (3), very good (4), excellent (5).

Never (1), rarely (2), sometimes (3), mostly (4), always (5).

NB these scores are reversed for negatively phrased items.

#### Rasch analysis of the cognition & fatigue factor

**Figure 4:**
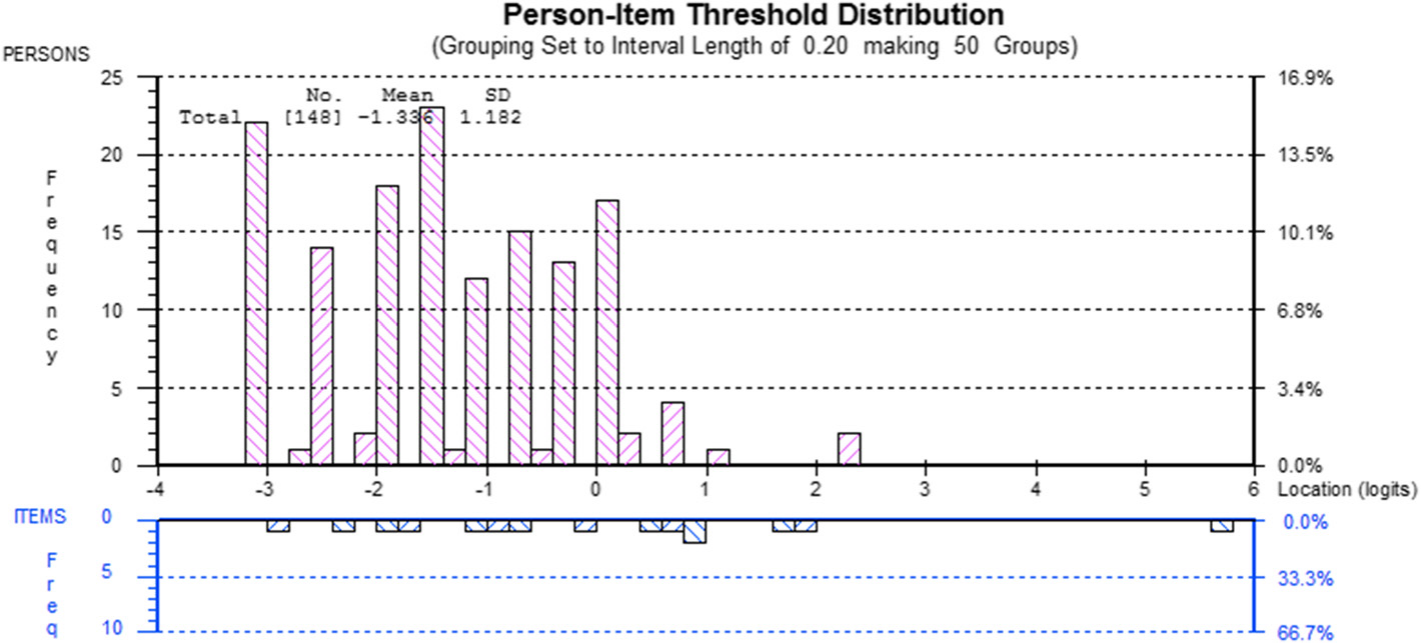
**Person-item map for the Cognition & Fatigue factor.**

The Cognition & Fatigue factor fitted the Rasch model (χ2 = 19.37, df = 12, p = 0.079). The fit residual SDs for both items and person were 1.15 and 0.86 respectively, indicting fit to the Rasch model. Threshold disordering was identified for items 13, 17 and 19 (Additional file 2). Twenty-two misfitting persons, of the 183 analysed, were also identified and subsequently removed from the analysis. The PSI was 0.659 indicating average internal consistency of the factor. DIF was not observed for any of the person factors. Two separate analyses were undertaken in order to achieve fit to the Rasch model. Fit to the Rasch model was achieved (χ2 = 15.82, df = 8, p = 0.045) by removing items 17 and 19 and 13 misfitting persons - these persons were not significantly different from the analysed persons with regard to demographics. Rescoring of item 13 resolved the threshold disordering (Additional file 2). The PSI was 0.611 and the fit residual SDs were 1.18 for items and 0.88 for persons. The items and scoring structure for the revised factor are presented in Additional file 3 with the item fit statistics at Table 3. There was no DIF for any person factor nor were there any residual correlations. The paired t-test between the positively and negatively loaded items on the Rasch factor in the PCA was significantly different indicating a unidimensional subscale. All items on this factor require rescoring prior to summing the total score. The total score for this factor is 19. The person-item distribution has a mean of −1.336 reflecting the negatively worded items on this factor (Figure 4).

The lack of DIF for any of the person factors on both subscales supports the construct validity of the 13-item PPM-O [21].

### Confirming the structure of the two-factor, 13-item PPM-O

**Figure 5:**
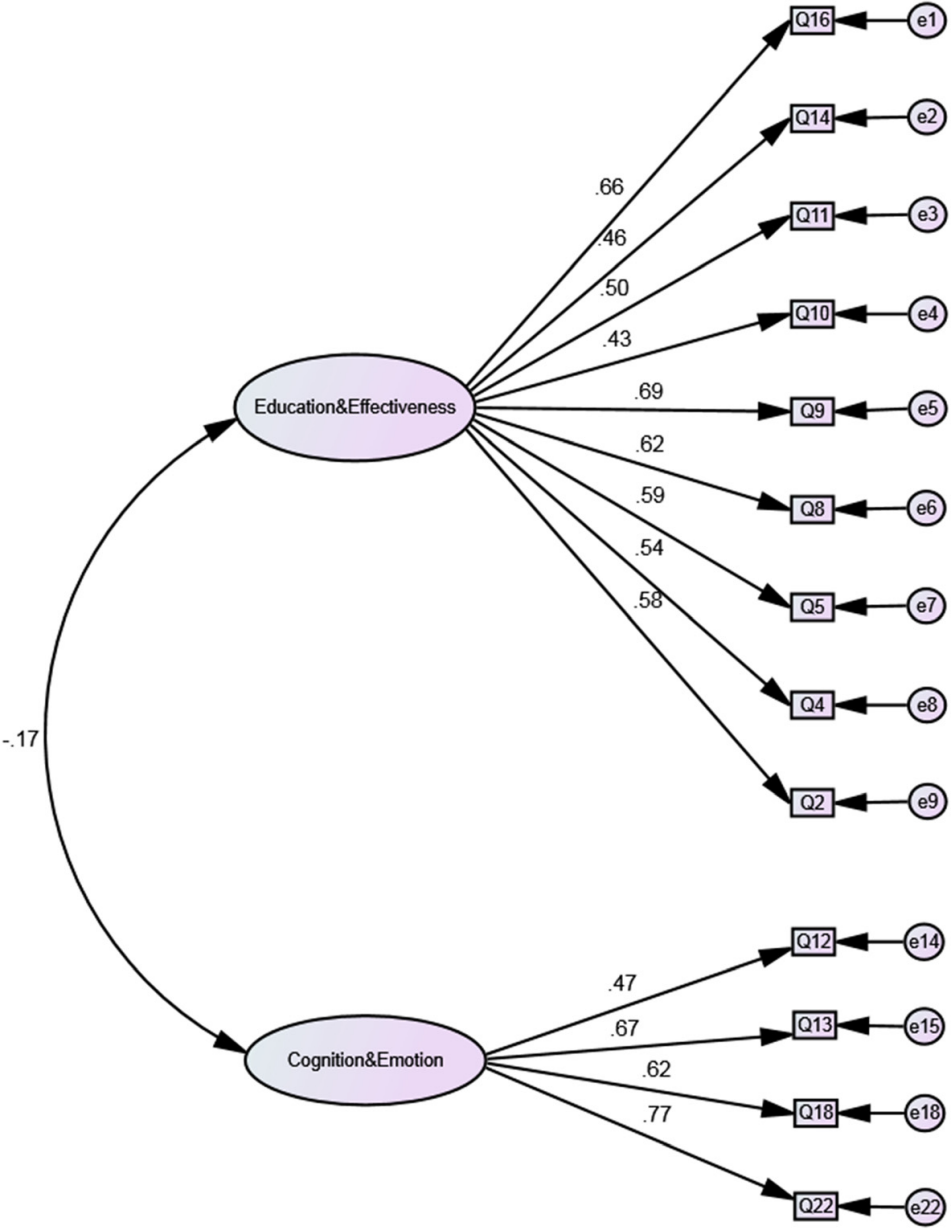
**Confirmatory factor analysis path diagram for the 2-factor, 13-item Patient Perception Measure -Osteopathy.**

The Rasch analysed two-factor PPM-O was then analysed with a CFA. The path diagram for the revised 13-item PPM-O is presented in Figure 5 and the model fit statistics are presented in Table 1. The negative association between the two factors (−0.17) supports the fact the PPM-O is multidimensional and that a total score for PPM-O should not be calculated. Rather a score for each of the factors should be calculated.

## Discussion

The purpose of the present study was to investigate the construct validity of the Patient Perception Measure – Osteopathy. The study used both classical test theory (CTT) and modern test theory (MTT) to investigate the properties of the questionnaire. The focus of the discussion is the CTT and MTT results rather than the descriptive statistics derived from the completed questionnaires.

### Psychometrics

The initial phase of the current research involved the analysis of the data set using confirmatory factor analysis (CFA). CFA is appropriate given that the *a-priori* domain structure of the PPM-O had previously been hypothesised by Mulcahy et al. [13]. The results of the CFA suggest that the domain structure proposed by these authors does not fit the data in the present study. Such a result would suggest that another subscale structure is more appropriate. Subsequent analysis using the Rasch model indicated substantial issues with a range of items and a multidimensional structure. The MTT and CTT results challenged the construct validity of the 22-item PPM-O suggesting further analysis was required.

Drawing on the results from the first CFA, modifications were made to the 22-item PPM-O in order to develop a subscale structure that fitted the data obtained in the present study. A two-factor, 15 item measure was produced and Rasch-analysed. To ensure that as many items as possible were retained to capture the patient experience with osteopathic treatment, both the Education & Effectiveness and Cognition & Fatigue subscales were Rasch-analysed separately. In essence, this produced two unidimensional subscales within the PPM-O. Following these Rasch analysis, a 2-factor, 13-item questionnaire was developed. It is important to note that given both subscales are unidimensional, they cannot be added together to form a single score for the PPM-O. This result is also supported by the CFA of the 13-item questionnaire where the data fitted the model and the correlation between the factors was negative. Support for the construct validity of the 13-item PPM-O is demonstrated by the lack of DIF for any of the items, that is, the response to an individual item was not affected by gender, age, satisfaction with life and meaningful daily activity.

The 13-item PPM-O measures two dimensions of the patients’ experiences of osteopathic treatment: information, education and effectiveness of treatment; and cognitive changes and fatigue experienced post-osteopathic treatment.

### Education & effectiveness factor

In the physical and manual therapy research, patients’ perceptions and experiences of treatment, information and education are often associated with favourable patient treatment outcomes [29-31] and meeting the patient’s expectations [11]. These aspects are also strongly represented in the patient satisfaction literature in physical therapy [3,6,32,33]. Given the literature identified here, the fact that information, education and effectiveness of treatment are contained in one dimension of the PPM-O in the current study is not surprising. Of note however is that previous measures have not included (i) Information, (ii) Education and (iii) Effectiveness into one dimension as has been demonstrated in the 13-item PPM-O. The items within this factor provide the practitioner with an overview of the effectiveness of their treatment, and suggest that there is a strong relationship between these three aspects of the patients’ perception of their treatment. Patient responses to the individual items in the Education & Effectiveness factor may assist practitioners in their future treatment and management of individual patients - if an individual patient’s ratings on a specific aspect of osteopathic care was not rated as being adequate or satisfactory by the patient, these aspects of care may be addressed by the clinician in future treatment.

To the authors’ knowledge, the PPM-O is the first self-report measure to evaluate the patients’ perception of the influence of the osteopathic principles on their treatment. Items 6 and 7 capture two of these principles [34]: 1) The human being is a dynamic unit of function (item 6), and 2) The body possesses self-regulatory mechanisms that are self-healing in nature. Cotton [35] contends that without using the principles “…osteopathy ceases to exist as a distinctive form of healthcare.” The presence of the two PPM-O items related to the osteopathic principles may assist in ascertaining whether the patient perceives osteopathy to be a “…distinctive form of healthcare”.

### Cognition & fatigue factor

Perceived change in cognitive function and fatigue associated with osteopathic treatment are captured in the PPM-O. Clinicians do not routinely assess cognitive effects of manual therapy, and there are no valid and reliable measures to assess these treatment outcomes. However, the PPM-O Cognition & Fatigue factor provides an avenue to explore these aspects of osteopathic treatment. The concept of assessing fatigue in the PPM-O is supported by its presence in the SF-36 [36]. The PPM-O may be used, albeit with caution at this stage, to assess osteopathic patients’ perceived effect of their treatments on cognitive functioning. Changes in cognitive function have been demonstrated in intensive care [37,38] and anaesthesia settings [39] however there is no support in the manual therapy literature for these post-treatment responses. This may be of some interest as the factor relates to the ability of patients to focus on tasks, concentrate, and feeling vague post osteopathic treatment, and could be the focus of subsequent studies. If patients frequently experience these cognitive responses to osteopathic treatment, clinicians should consider them when treating and managing their patients. In particular care should be taken to ensure that a patient is sufficiently alert and capable of performing their daily activities post-treatment. Consequently testing of cognitive responses to osteopathic treatment is required. To further explore cognitive responses to osteopathic treatment, future research may also include a pre- and post- treatment testing of visual or auditory attention, problem solving, or short-term memory [38].

### Future opportunities

From a practical standpoint, the PPM-O is a brief 13-item measure and would take less than five minutes for patients to complete. Clinicians and educators can score and analyse the measure in less than five minutes. Subsequently the implementation of the questionnaire in both research and clinical practice settings is feasible. Users of the PPM-O should be aware that it is not designed to have a total questionnaire score calculated. Rather, the total score for each factor should be calculated separately using the questionnaire interpretation provided in Additional file 3.

Prior to using the PPM-O in practice or research settings, the concurrent validity of the items in the Education & Effectiveness factor needs to be assessed against other patient satisfaction measures such as the measure developed by Hawthorne et al. [9]. Assessment of the concurrent validity of the Cognition & Fatigue factor items may prove more challenging, and may require a range of measures of cognitive functioning to be employed. Identifying the presence of depression, anxiety or other conditions that may affect vitality and energy is also recommended.

### Limitations

A limitation of the study is the population who participated in the study. These patients were attending Australian osteopathy university-based teaching clinics and may not necessarily be representative of the population who attend for private osteopathic care [40]. Further work is required to investigate the validity of the PPM-O in a private osteopathic patient population. In addition, the removal of approximately 18% of the questionnaires from the data set to be analysed in the CFA may have introduced bias into the results. No analysis of the questionnaires that were removed prior to the analysis was undertaken therefore it is not possible to comment on how the responses to these questionnaires may have impacted upon the results. The decision to remove incomplete questionnaires was made *a priori* as it was felt this would be more efficient for the CFA. The characteristics of the patients who did not complete all items on the PPM-O and the demographic questionnaire were analysed to ascertain whether this population differed from those who completed all items. There were no differences between these groups, however there was no record of the number of questionnaires provided to patients and therefore a count of those potentially not returned for analysis was not possible. Further, no data were collected as to why any patients chose not to complete the PPM-O and this may have introduced some bias into the study.

## Conclusions

The present study has modified a measure of patients’ perception of their osteopathic treatment (the PPM-O) and, in part, established the construct validity of the modified measure through the use of both CTT and MTT. The use of both of these statistical approaches has developed a measure with strong psychometric properties across the two factors in the PPM-O13. Currently the PPM-O provides a measure of the patient-therapist interaction, including information, education and effectiveness, as well as a potential measure of cognitive functioning and fatigue experienced during, and after, osteopathic treatment. The items in the Education & Effectiveness factor are consistent with previous literature, however the inclusion of items that relate to the osteopathic principles in a PROM is described in the literature for the first time. The patients’ perception of osteopathy principles has not previously been explored and provides an interesting avenue for future research. The cognitive and fatigue aspects related to treatment outcomes has received little attention in the manual and physical therapy literature, and on the basis of the observations made in this study warrants further investigation.

## Additional files

Additional file 1:
**PPM-O Education & Effectiveness Factor – Category Probability Curves.**
Additional file 2:
**PPM-O Cognition & Fatigue Factor – Category Probability Curves.**
Additional file 3:
**PPM-O scoring guide.**
