# Supplementary material for: Exploring the construct validity of the Patient Perception Measure – Osteopathy (PPM-O) using classical test theory and Rasch analysis
Source: Chiropr Man Therap. 2015 Mar 2;23:6. doi: 10.1186/s12998-015-0055-x (PMC4346115; doi:10.1186/s12998-015-0055-x)
Supplement: Additional file 2: — PPM-O Cognition & Fatigue Factor – Category Probability Curves. [file 12998_2015_55_MOESM2_ESM.docx]

**PPM-O Cognition & Fatigue Factor – Category Probability Curves (initial analysis)**


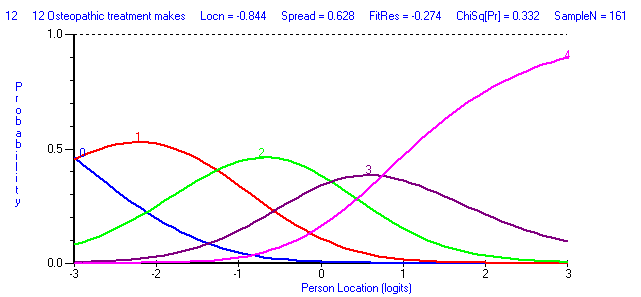


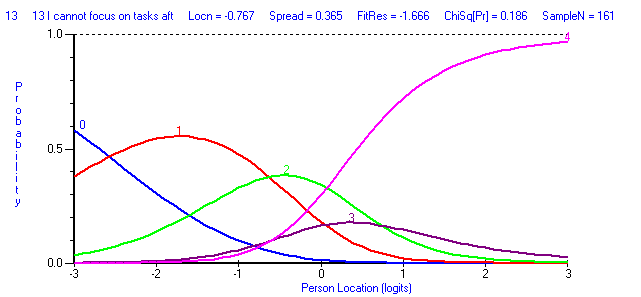


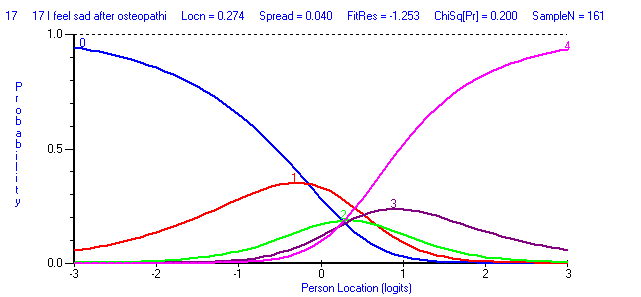


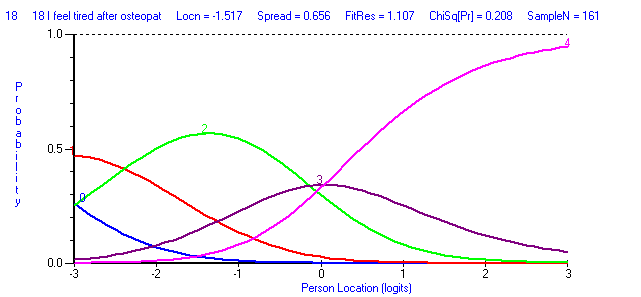


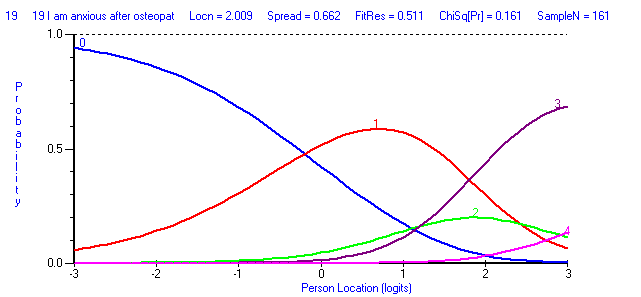


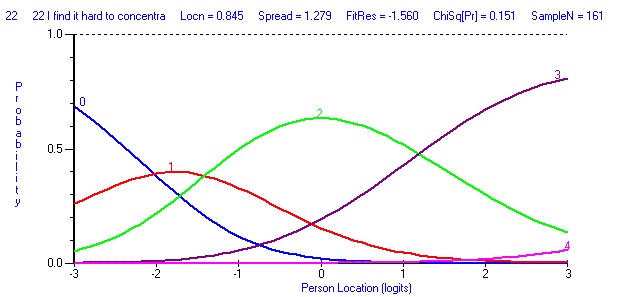


**PPM-O Cognition & Fatigue Factor – Category Probability Curves (final analysis with rescoring)**

**
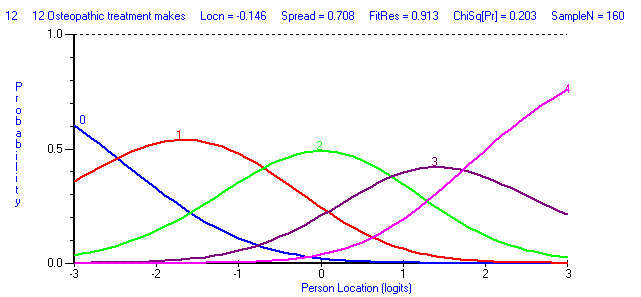

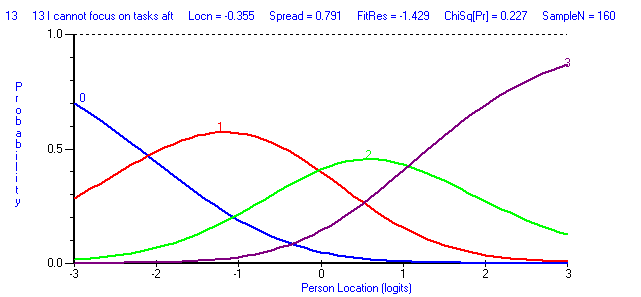

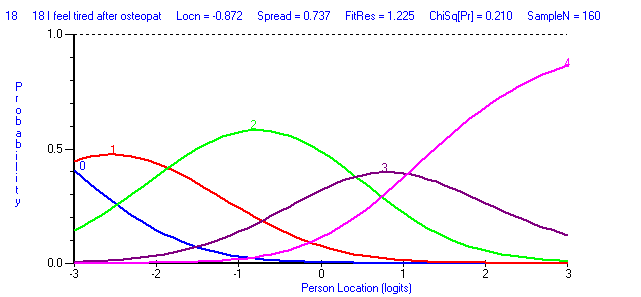

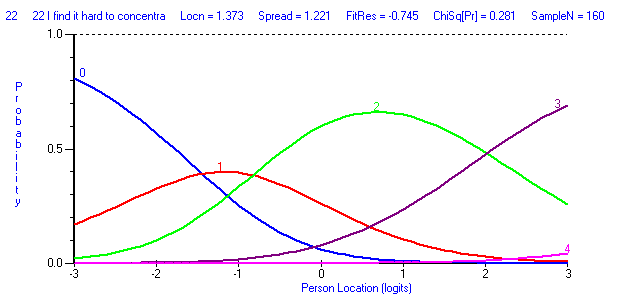
**
