# Supplementary material for: Exploring the construct validity of the Patient Perception Measure – Osteopathy (PPM-O) using classical test theory and Rasch analysis
Source: Chiropr Man Therap. 2015 Mar 2;23:6. doi: 10.1186/s12998-015-0055-x (PMC4346115; doi:10.1186/s12998-015-0055-x)
Supplement: Additional file 3: — PPM-O scoring guide. [file 12998_2015_55_MOESM3_ESM.docx]

|  | **Scale options** | | | | |
| --- | --- | --- | --- | --- | --- |
| **Education & Effectiveness** | 1 | 2 | 3 | 4 | 5 |
| 1. The way my osteopath answers all of my questions is | 1  Poor | 1  Fair | 2  Good | 3  Very good | 4  Excellent |
| 2. The instructions my osteopath gives me regarding my home exercise program are | 1  Poor | 1  Fair | 2  Good | 3  Very good | 4  Excellent |
| 3. Osteopathic treatment has helped my condition | 1  Never | 1  Rarely | 2  Sometimes | 3  Mostly | 4  Always |
| 4. As a result of osteopathic treatment, my general health is | 1  Poor | 2  Fair | 3  Good | 4  Very good | 5  Excellent |
| 5. During my treatment, the questions my osteopath asked were | 1  Poor | 1  Fair | 2  Good | 3  Very good | 4  Excellent |
| 6. After my osteopathic treatment I felt like my whole body was treated rather than just one area | 1  Never | 2  Rarely | 3  Sometime | 4  Mostly | 5  Always |
| 7. Osteopaths at this clinic talk about the body's ability to heal itself | 1  Never | 1  Rarely | 2  Sometimes | 3  Mostly | 4  Always |
| 8. I feel calmer after my osteopathic treatment | 1  Never | 1  Rarely | 2  Sometimes | 3  Mostly | 4  Always |
| 9. How helpful is osteopathic treatment in managing your condition | 1  Poor | 2  Fair | 3  Good | 4  Very good | 5  Excellent |
| **Cognition & Emotion** |  |  |  |  |  |
| *10. Osteopathic treatment makes me feel vague* | 1  Never | 2  Rarely | 3  Sometimes | 4  Mostly | 5  Always |
| *11. I cannot focus on tasks after my osteopathic treatment* | 1  Never | 2  Rarely | 3  Sometimes | 4  Mostly | 4  Always |
| *12. I feel tired after osteopathic treatment* | 1  Never | 2  Rarely | 3  Sometimes | 4  Mostly | 5  Always |
| *13. I find it hard to concentrate after my osteopathic treatment* | 1  Never | 2  Rarely | 3  Sometimes | 4  Mostly | 5  Always |

Note: Negatively phrased items are in italics and require rescoring prior to analysis.
